# Highly Pathogenic Avian Influenza Virus Nucleoprotein Interacts with TREX Complex Adaptor Protein Aly/REF

**DOI:** 10.1371/journal.pone.0072429

**Published:** 2013-09-20

**Authors:** Vinod R. M. T. Balasubramaniam, Tham Hong Wai, Bimo Ario Tejo, Abdul Rahman Omar, Sharifah Syed Hassan

**Affiliations:** 1 Virus-Host Interaction Group, Infectious Disease Laboratory (MR3), School of Medicine and Health Sciences, Monash University Sunway Campus, Selangor, Malaysia; 2 Department of Chemistry, Faculty of Science, University Putra Malaysia (UPM) Serdang, Selangor, Malaysia; 3 Institute of Bioscience, University Putra Malaysia (UPM) Serdang, Selangor, Malaysia; Centers for Disease Control and Prevention, United States of America

## Abstract

We constructed a novel chicken (*Gallus gallus*) lung cDNA library fused inside yeast acting domain vector (pGADT7). Using yeast two-hybrid screening with highly pathogenic avian influenza (HPAI) nucleoprotein (NP) from the strain (A/chicken/Malaysia/5858/2004(H5N1)) as bait, and the *Gallus gallus* lung cDNA library as prey, a novel interaction between the *Gallus gallus* cellular RNA export adaptor protein Aly/REF and the viral NP was identified. This interaction was confirmed and validated with mammalian two hybrid studies and co-immunoprecipitation assay. Cellular localization studies using confocal microscopy showed that NP and Aly/REF co-localize primarily in the nucleus. Further investigations by mammalian two hybrid studies into the binding of NP of other subtypes of influenza virus such as the swine A/New Jersey/1976/H1N1 and pandemic A/Malaysia/854/2009(H1N1) to human Aly/REF, also showed that the NP of these viruses interacts with human Aly/REF. Our findings are also supported by docking studies which showed tight and favorable binding between H5N1 NP and human Aly/REF, using crystal structures from Protein Data Bank. siRNA knockdown of Aly/REF had little effect on the export of HPAI NP and other viral RNA as it showed no significant reduction in virus titer. However, UAP56, another component of the TREX complex, which recruits Aly/REF to mRNA was found to interact even better with H5N1 NP through molecular docking studies. Both these proteins also co-localizes in the nucleus at early infection similar to Aly/REF. Intriguingly, knockdown of UAP56 in A549 infected cells shows significant reduction in viral titer (close to 10 fold reduction). Conclusively, our study have opened new avenues for research of other cellular RNA export adaptors crucial in aiding viral RNA export such as the SRSF3, 9G8 and ASF/SF2 that may play role in influenza virus RNA nucleocytoplasmic transport.

## Introduction

A ‘‘host cellular factory’’ has thousands of machines, which viruses co-opt or subvert for each step of their life cycle. Generally, viruses initiate their life cycle by attaching to host cell surface receptors, entering the cells, uncoating the viral nucleic acid, and replicating their genome. After new copies of viral proteins and genes are synthesized, these components assemble into progeny virions, which exit the cell [1]. So, it is best summarized that the host cellular machinery provides an important platform for the survival of many viruses. For example, the nucleus is a crucial site of replication for a select group of viruses such as the herpes, adeno-, influenza and retroviruses. Thus, a proper regulated mechanism for the nucleocytoplasmic transport of these viral nucleic acids is vital to begin and complete the viral replicative cycle. Influenza virus genome consists of segmented negative sense RNA which are bound by the viral polymerase at each terminus, coated with nucleoprotein forming viral ribonucleoprotein (RNP) complex. In infected cells, the influenza virus is uncoated and delivers its RNP into the cytoplasmic matrix. The RNP is then transported into nucleus for the viral replicative cycle to begin [2]. Influenza virus nucleoprotein (NP) presents an interesting avenue for research as it shuttles between the nucleus and the cytoplasm during infection. Apart from that, it is most abundantly expressed protein during the course of infection with multiple functionalities [3]. A nuclear localization signal (NLS) has also been identified in NP at amino acids 327 to 345 [4], not forgetting also its primary function which is viral genome encapsidation [5]. NP accumulates in the nucleus in the early phases of infection and is exclusively distributed in cytoplasm later during viral assembly and maturation [6]. Whereas the functions of the influenza viral proteins have been studied extensively during the last decade, relatively little is known about the cellular factors involved in influenza virus life cycle.

We were interested in identifying new cellular interactors of NP from a highly virulent A/H5N1 bird-flu isolate {A/chicken/Malaysia/5858/2004 H5N1}, which may facilitate viral replication. It would be really interesting to uncover novel cellular interactors from the primary host of the virus. For this reason, we constructed a novel chicken (*Gallus gallus*) lung cDNA library fused inside yeast acting domain vector (pGADT7). The library was checked for its titer, complexity and quality. Following that, H5N1 NP (Gen Bank accession no.84797647) of the Malaysian isolate was used as bait to search for novel interactors in a yeast two-hybrid system based screen of chicken lung cDNA library. In the screen, we identified that H5N1 NP interacts with *Gallus gallus* THO complex 4 (THOC4) or Aly/REF, Gen Bank accession no. 363740920. Aly/REF as one of the major cellular RNA export adaptor protein [7], and its interaction with influenza A NP never have been reported before. We validated this interaction using mammalian two hybrid assay and also using simple co-immunoprecipitation assay shown in Figure S1. Confocal microscopy studies in Vero cells revealed that NP co-localizes with Aly/REF in the nucleus at early infection. Molecular docking studies using crystal structures from Protein Data Bank suggests that human Aly/REF interacts favorably with NP gene of influenza virus A/HK/483/97(H5N1). In conjunction with this, we performed mammalian two hybrid assay on NP of different subtypes of influenza A which is the swine A*/*NewJersey*/*1976*/*H1N1 and pandemic A/Malaysia/854/2009(H1N1) with human Aly/REF. Results show strong interaction between these proteins. NP from these viruses also co-localizes in the nucleus of infected A549 cells. To assess the role of Aly/REF in viral RNA export, small interfering RNA designed to knockdown this factor was used. However, knockdown of Aly/REF had little effect on the export of NP and other viral RNA as there were no significant reductions in virus yields in knockdown infected cells. Interestingly, knockdown of UAP56, another member of the TREX complex showed significant reduction in viral titer in influenza infected cells, suggesting the possible role played by this complex in nucleocytoplasmic transport of viral mRNA.

## Results

### Construction of novel chicken lung cDNA library

**Figure 1:**
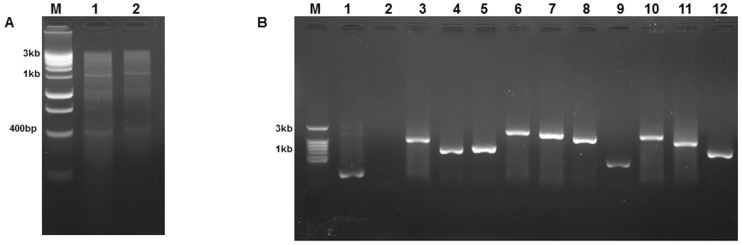
High-quality double-stranded cDNA generated using SMART™ cDNA synthesis and library complexity check *via* colony PCR amplification on randomly picked yeast colonies. Figure 1A illustrates the agarose gel of total cDNA generated from whole lung of 4 weeks old specific pathogen free (SPF) white leghorn chicken using the Make Your Own “Mate & Plate” Library System. Lane M: 1kb ladder molecular weight standard. Lane 1: Purified total chicken lung cDNA before size selection with CHROMA SPIN + TE-400 columns. Lane 2: Reduced abundance of purified cDNA below 400 bp compared to lane 1 after selection with CHROMA SPIN + TE-400 columns. Figure 1B shows the agarose gel of yeast colony PCR screenings of 10 colonies revived from a 1-ml stock of cDNA library cultured on SD/-Leu plates. Inserts of different sizes indicate the complexity of the library created. Lane M: 1 kb ladder molecular weight standard. Lane 1: positive control using pGADT7 vector as template, Lane 2: negative control. Lane 3 to 12: 10 randomly picked yeast recombinant clones from the constructed chicken lung cDNA library.

A novel *Gallus gallus* cDNA library for use in the yeast-two-hybrid system was constructed. Total RNA of lungs from 4 weeks old specific pathogen free (SPF) white leghorn chicken was extracted and converted into double-stranded cDNA (ds-cDNA) using SMART™ cDNA synthesis technology (Clontech). Purified ds-cDNA pool was then co-transfected with linearized pGADT7-rec vector into competent yeast *Saccharomyces cerevisiae* strain Y187. Double-stranded cDNA less than 400 bp was discarded using Chroma Spin™ TE-400 Columns (Clontech) as they can be unwanted products of incomplete first and second-strand synthesis (Figure 1A). Apart from that, this step increases the chance of isolating full-length cDNA encoding large proteins. The constructed library was calculated for its titer and showed that it can produce 6×10^7^ yeast colony forming unit/ml. Library complexity was also checked by using 10 randomly picked colonies from a SD/-Leu plate and amplification of inserts was done using 2× Terra™ PCR Direct Red Dye Premix (Clontech) with the Insert Check primers (Clontech) (Table 1). Figure 1B shows the average size and complexity of cDNA inserts in the library. Overall, a high quality cDNA library from whole lung of white leghorn chicken was generated for the use in yeast two hybrid.

**Table 1:** Insert Check primers (Clontech).

Primer Name	Sequences (5′–3′)
Insert Check forward	CTATTCGATGATGAAGATACCCCACCAAACCC
Insert Check reverse	GTGAACTTGCGGGGTTTTTCAGTATCTACGATT

### Identification of *Gallus gallus* THO complex 4 (THOC4)/Aly/REF as an interacting partner of HPAI H5N1 NP

The constructed *Gallus gallus* cDNA library was screened using H5N1 NP as bait in GAL4 based Matchmaker yeast two-hybrid system (Clontech). Yeast cells were transformed with bait (Y2H Gold strain) and prey (Y187 strain) plasmids and selected for growth on selective leucine, tryptophan, adenine and histidine (-Leu, -Trp, -Ade, -His) plates supplemented with Aureobasidin A and X-α-gal. β-galactosidase positive colonies on quadruple dropout medium were further analyzed (example of positive colonies are showed in Figure S2). Plasmid from positive colonies were isolated and subjected to DNA sequencing followed by BLASTn analysis to identify their cDNA insert. The *Gallus gallus* THO complex 4 (THOC4) or Aly/REF, Gen Bank accession no. 363740920 was identified as an interacting partner of H5N1 NP.

### Transiently expressed H5N1 NP interacts with *Gallus gallus* THO complex 4 (THOC4)/Aly/REF in mammalian cells

**Figure 2:**
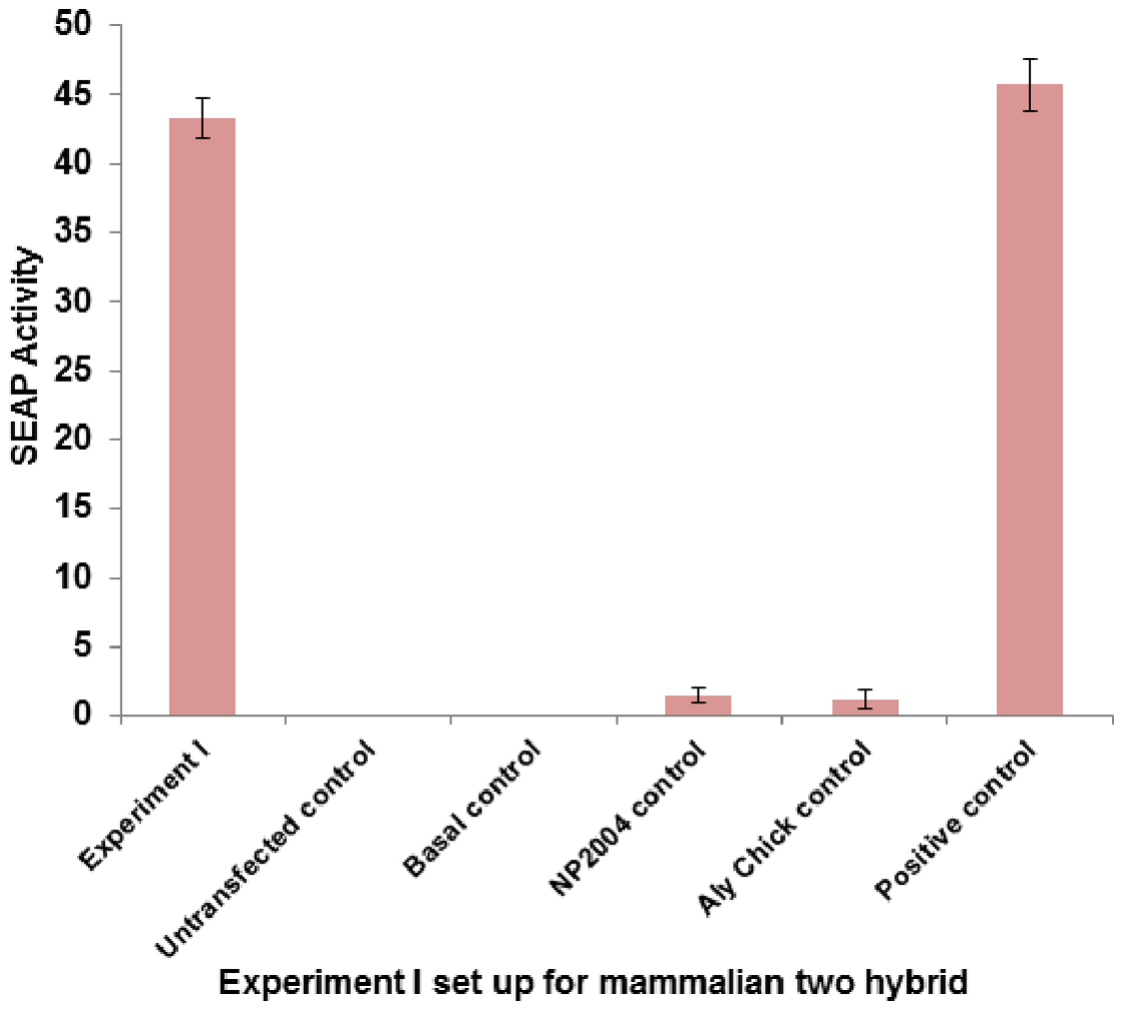
Confirmation of physical interaction between NP of Influenza A/chicken/Malaysia/5858/2004 virus with *Gallus gallus* THO complex 4 (THOC4) or Aly/REF (Experiment 1). Mammalian two hybrid assay was conducted in 24-well plate. HEK293T cells were transfected with respective plasmids (Table 2) in triplicates, incubated at 37°C, 5% CO_2_, for 48 hours. Culture media were harvested and tested for SEAP activity using GreatEscAPe™ SEAP Chemiluminescence Detection Kit (Clontech). The values reported are averages from 3 independent transfections (±S.D). SEAP activity directly reflects the interaction between H5N1 NP and *Gallus gallus* THO complex 4 (THOC4). Results show that NP-Aly/REF interaction was relatively strong and statistically comparable (p-value >0.05) to when compared to the positive control pM3-VP16.

NP-Aly/REF interaction was further validated using mammalian two hybrid assay. Both the coding sequence for NP (A/chicken/Malaysia/5858/2004) H5N1 and *Gallus gallus* Aly/REF were subcloned into pM (GAL4 DNA-BD) and pVP16 (AD) cloning vectors (Clontech), respectively. These vectors were co-transfected along with a pG5SEAP reporter vector into HEK293T cells using CalPhos™ Mammalian Transfection Kit (Clontech) (Table 2). pG5SEAP, when transcriptionally activated by physical interaction between pM and pVP16 conjugated proteins, expresses SEAP (secreted alkaline phosphatase). The SEAP activity was determined 48 h following transfection, and detected by4-methylumbelliferyl phosphate fluorescence at 360 nm (Full experimental procedure is described under “Materials and Methods” section). The SEAP reporter gene encodes a truncated form of the placental enzyme without the membrane anchoring domain. As a result, the protein is secreted from the transfected cells into the culture medium. Therefore, the level of SEAP activity is a direct measure of protein-protein interaction because it is directly proportional to changes in intracellular concentration of SEAP mRNA and proteins [8]. Results show that NP-Aly/REF interaction was relatively strong, when compared to the positive control pM3-VP16 (Figure 2).

**Table 2:** Set-up for mammalian-two hybrid assays.

Transfection	GAL4 DNA-BD Plasmid	VP16 AD Plasmid	Reporter Plasmid
Experiment I	pM_NP2004	pVP16_AlyChick	pG5SEAP
Experiment II	pM_NP1976	pVP16_Aly Human	pG5SEAP
Experiment III	pM_NP2009	pVP16_Aly Human	pG5SEAP
Untransfected control	None	None	None
Basal control	pM	pVP16	pG5SEAP
NP2004 control	pM_NP2004	pVP16	pG5SEAP
NP1976 control	pM_NP1976	pVP16	pG5SEAP
NP2009 control	pM_NP2009	pVP16	pG5SEAP
Aly Chick control	pM	pVP16_Aly Chick	pG5SEAP
Aly Human control	pM	pVP16_Aly Human	pG5SEAP
Positive control	pM3-VP16	pM3-VP16	pG5SEAP

### Molecular docking using crystal structures of human THOC4 or Aly/REF (PDB: 3ULH) and H5N1 nucleoprotein (PDB: 2Q06)

**Figure 3:**
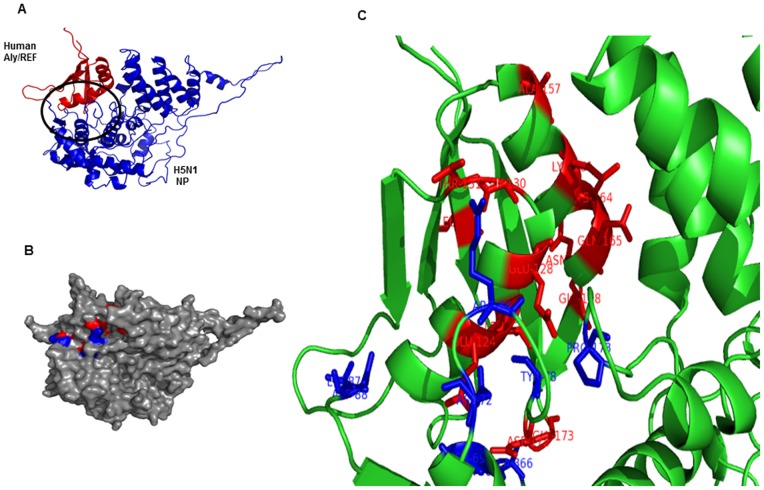
Molecular docking of human THOC4 or Aly/REF (PDB: 3ULH) and H5N1 nucleoprotein (PDB: 2Q06). Figure 3A shows ribbon representation of docking between H5N1 NP and human Aly/REF. Both the molecules shows favorable and tight binding with binding energy of −22.81 kcal/mol. Figure 3B shows the surface representation of the both interacting molecules. Aly/REF (3ULH) residues that interact with H5N1 nucleoprotein (2Q06) are red, and H5N1 nucleoprotein (2Q06) residues that interact with Aly/REF (PDB: 3ULH) are blue. Amino acid residues of 2Q06 that directly interact with 3ULH are, Ser69, Asp72, Arg74, Tyr78, Lys87, Asp88, Pro173, Ala366, and Ser367. Meanwhile, 3ULH residues that interact with 2Q06 are Leu119, Glu124, Leu125, Glu128, Gly130, Thr131, Ala157, Lys161, Lys164, Gln165, Asn167, Gly168, Asp172, and Gly173. Magnified version of the interacting residues are shown in Figure 3C.

It would be interesting to investigate whether this interaction is also predominant in human Aly/REF. We conducted molecular docking studies using three-dimensional structure of human THOC4 or Aly/REF (PDB: 3ULH) and H5N1 nucleoprotein (PDB: 2Q06). The full experimental procedure for docking is explained clearly in the “Materials and Methods” section. Overall, both proteins interact strongly with a favorable binding energy of −22.81 kcal/mol. There are several amino acid residues of 2Q06 that directly interact with 3ULH. They are, Ser69, Asp72, Arg74, Tyr78, Lys87, Asp88, Pro173, Ala366, and Ser367. Meanwhile, 3ULH residues that interact with 2Q06 are Leu119, Glu124, Leu125, Glu128, Gly130, Thr131, Ala157, Lys161, Lys164, Gln165, Asn167, Gly168, Asp172, and Gly173. Figure 3 show how both protein interacts. In ribbon presentation, 3ULH is red and 2Q06 is blue (Figure 3A). In surface representation, 3ULH residues that interact with 2Q06 are red, and 2Q06 residues that interact with 3ULH are blue (Figure 3B). Figure 3C shows the magnified version of the interacting residues.

### H5N1 NP from different subtypes interacts with human THO complex 4 (THOC4)/Aly/REF in mammalian cells

**Figure 4:**
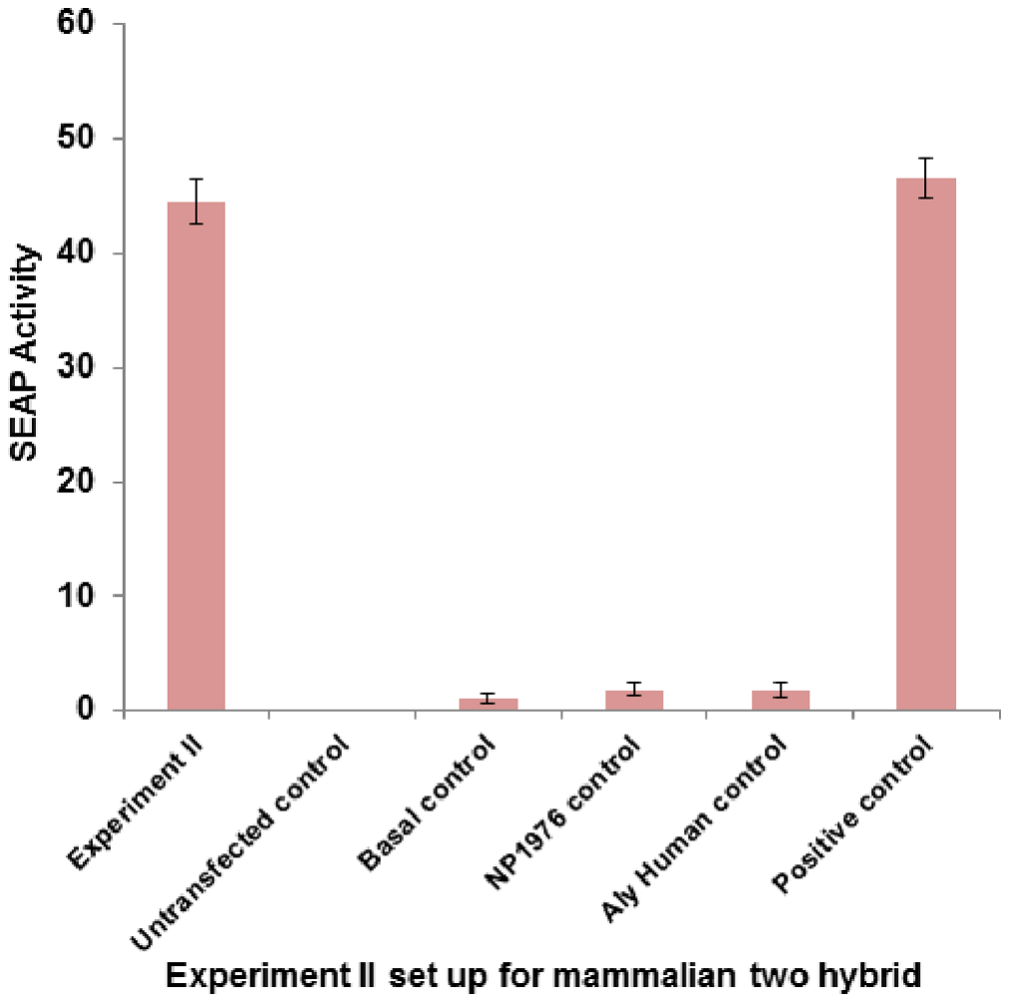
Interaction between NP of Influenza A/New Jersey/1976/H1N1 virus with human THO complex 4 (THOC4) or Aly/REF (Experiment II). Mammalian two hybrid assay was conducted in 24-well plate. HEK293T cells were transfected with respective plasmids (Table 2) in triplicates, incubated at 37°C, 5% CO_2_, for 48 hours. Culture media were harvested and tested for SEAP activity using GreatEscAPe™ SEAP Chemiluminescence Detection Kit (Clontech). The values reported are averages from 3 independent transfections (±S.D.). SEAP activity directly reflects the interaction between H1N1 NP and human THO complex 4 (THOC4). Results show that NP-Aly/REF interaction was relatively strong and statistically comparable (p-value >0.05) to when compared to the positive control pM3-VP16.

**Figure 5:**
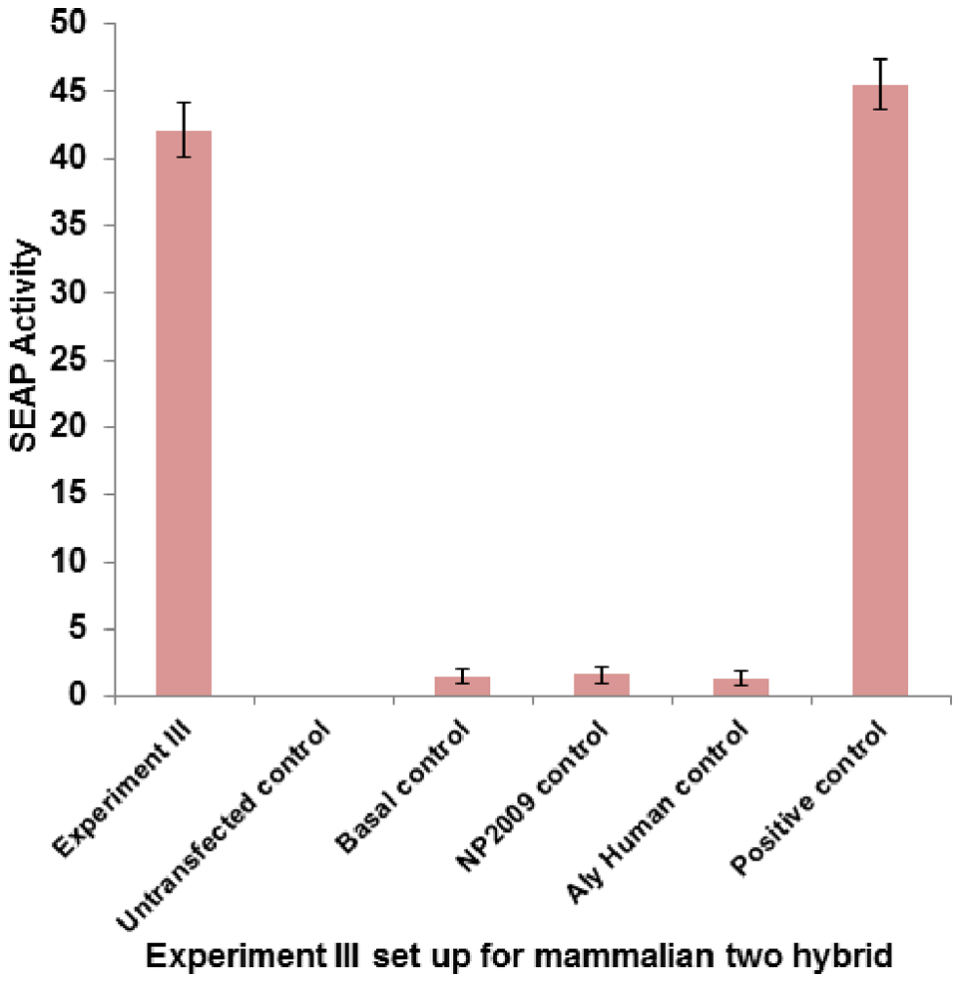
Interaction between NP of pandemic Influenza A/Malaysia/854/2009(H1N1) virus with human THO complex 4 (THOC4) or Aly/REF (Experiment III). Mammalian two hybrid assay was conducted in 24-well plate. HEK293T cells were transfected with respective plasmids (Table 2) in triplicates, incubated at 37°C, 5% CO_2_, for 48 hours. Culture media were harvested and tested for SEAP activity using GreatEscAPe™ SEAP Chemiluminescence Detection Kit (Clontech). The values reported are averages from 3 independent transfections (±S.D.). SEAP activity directly reflects the interaction between pandemic H1N1 NP and human THO complex 4 (THOC4). Results show that NP- human Aly/REF interaction was also relatively strong and statistically comparable (p-value >0.05) to when compared to the positive control pM3-VP16 and other 2 experiments conducted with different subtypes of Influenza.

We further validated our docking results by performing mammalian two hybrid assay. NP from the laboratory adapted A/New Jersey/1976/H1N1 Gen Bank accession no. 433698891 and pandemic A/Malaysia/854/2009(H1N1) Gen Bank accession no. 262118915 were subcloned into pM (GAL4 DNA-BD). Human Aly/REF Gen Bank accession no. 238776832 were also subcloned and pVP16 (AD) cloning vectors (Clontech). These vectors were co-transfected along with a pG5SEAP reporter vector into HEK293T cells (Table 2), just as described previously. SEAP activity was determined 48 h following transfection, and detected by 4-methylumbelliferyl phosphate fluorescence at 360 nm. Figure 4 and 5 shows the results of these experiments. NP from both these subtypes interacts favorably with human Aly/REF when compared to the positive control, consistent with our docking results. This further proves to show this interaction maybe conserved between influenza A species.

### H5N1 NP and Aly/REF co-localize primarily in the nucleus of mammalian cells

**Figure 6:**
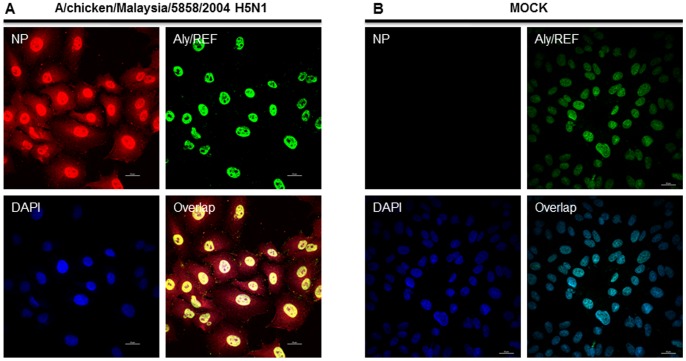
Co-localization of HPAI H5N1 NP from A/chicken/Malaysia/5858/2004 and Aly/REF in nucleus of mammalian cells. ***A and B*** Vero cells were seeded onto cover slips in a 24 well plate at a density of 10^4^/well. The cells are then infected with influenza A/chicken/Malaysia/5858/2004 virus at a multiplicity of infection (MOI) of 5 (***For A only***). A low (4°C) temperature pre-incubation method was used to allow synchronized infection for 1 h in DMEM medium supplemented with 2% BSA (GIBCO). After 1 h incubation, the cells were washed with DMEM once and then grown with DMEM supplemented with 0.2% BSA and 1 µg/ml N-p-tosyl-1-phenyl alanine chloromethyl ketone (TPCK) (Sigma Aldrich). At 12 hour p.i., cells were fixed and processed for immunostaining. NP was stained using anti-NP monoclonal primary antibody and Alexa594 conjugated secondary antibody (Red). Aly/REF was stained using Aly/REF specific primary antibody and Alexa488 conjugated secondary antibody (Green). Nuclei were stained with DAPI (Blue). ***A*** shows H5N1 infected cells whereas ***B*** shows control mock infected cells. Panels are labeled for their respective staining. Lower right panel in ***A*** shows primarily nuclear co-localization of NP and Aly/REF.

The distribution of Aly/REF in influenza virus infected cells was not studied before. Thus, we investigated the cellular localization pattern of H5N1 NP in context to Aly/REF in mammalian cells. First, we infected Vero cells with H5N1 (A/chicken/Malaysia/5858/2004) for 12 h, and an immunofluorescence staining was performed with specific antibodies. Results show that NP and cellular Aly/REF co-localize primarily in the nucleus (Figure 6). Similar results were obtained with A549 cells infected with the *New Jersey* and *pandemic* virus (results not shown). Collectively, our confocal microscopy studies have revealed that NP from these different subtypes and Aly/REF were present primarily in the nucleus at early infection.

### siRNA mediated inhibition of Aly/REF in H5N1 infected cells

**Figure 7:**
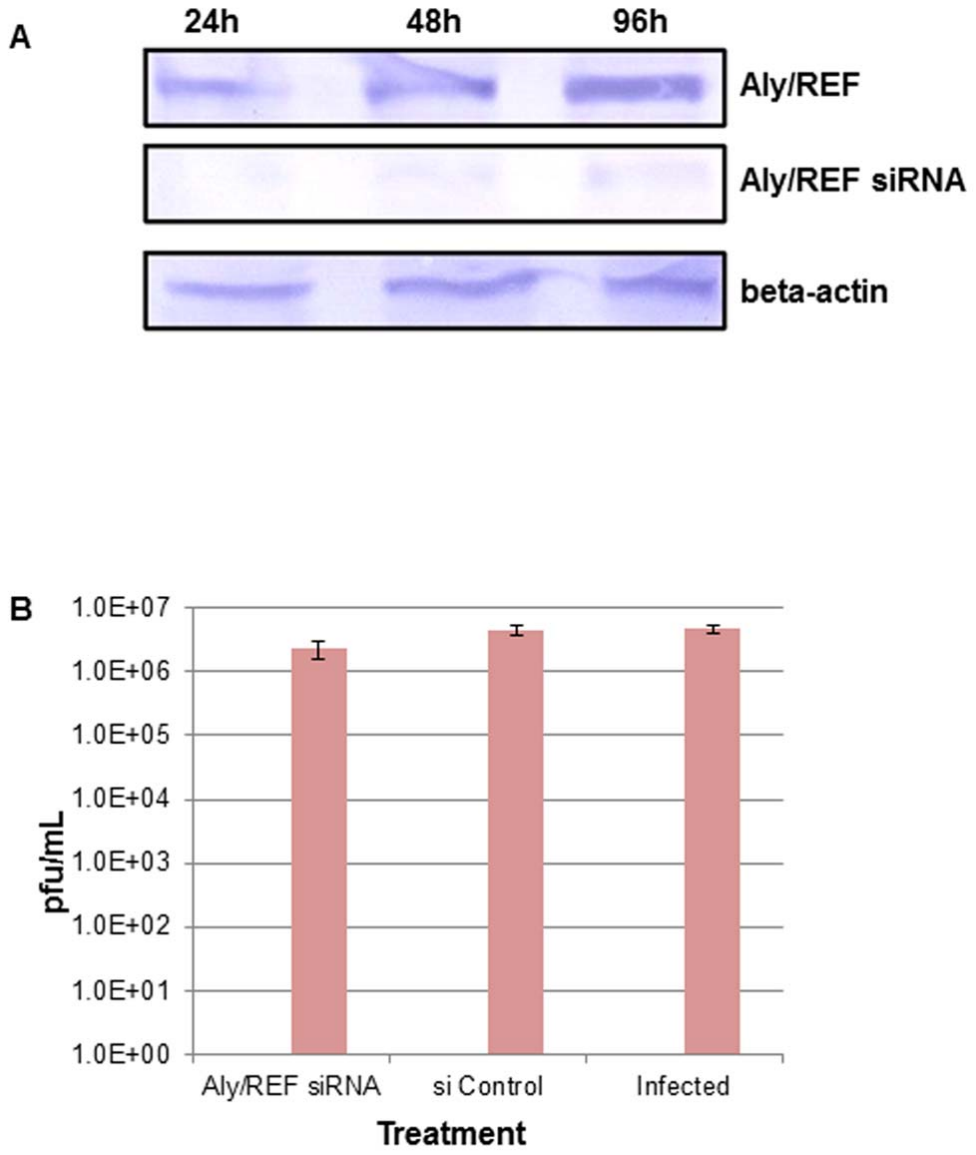
Knockdown of Aly/REF do not impair A/chicken/Malaysia/5858/2004 H5N1 virus growth. Figure 9A shows the knockdown efficiency, briefly, low-passage A549 cells were transfected with siRNA directed at Aly/REF at a concentration of 100 nM at varying time points; 24, 48 and 96 hour according to the manufacturer's instructions, and the knockdown efficiency was checked by Aly/REF specific antibody, beta actin was used as loading control. Figure 9B, A549 cells were transfected with siRNA targeting Aly/REF or with a non-targeting siRNA control as indicated. Fourty-eight hours later, depleted cells were infected with A/chicken/Malaysia/5858/2004H5N1 at MOI of 5. Cells were harvested 48 hours after infection and viral titers were determined by plaque assays on Vero cells. Overall, no significant reduction in virus yields was found in siRNA treated compared to non-treated infected cells.

We were also interested to determine whether the knockdown of Aly/REF would have any effect on the viral replication thus contributing to reduction of the overall viral yield. Gene-specific small interfering RNAs (siRNAs) for Aly/REF (Thoc4; 012078) provided as an On-Target Plus pools, which is a pool of four siRNAs targeting various sites in a single gene. The non-targeting siRNA 3 (siControl; D-001210) were provided as individual siRNA duplexes. All siRNAs were purchased from Dharmacon (Dharmacon Research Inc., Lafayette, CO) and transfected using Lipofectamine RNAiMAX (Invitrogen). Low-passage A549 cells were transfected with siRNA at a concentration of 100 nM for 48 hour according to the manufacturer' instructions, and the knockdown efficiency was checked by immunoblot analysis (Figure 7A). To study the effect of Aly/REF knockdown on the replication of influenza virus, A/chicken/Malaysia/5858/2004 H5N1 strain were used to infect the siRNA treated A549 cells at an MOI of 5. Supernatants were collected 48 hour p.i. and the virus titers were determined by plaque assay on Vero cells. Overall, the plaque assay result showed no significant reduction in virus yields in siRNA treated compared non-treated infected cells (Figure 7B).

### Molecular docking using crystal structures of human UAP56 (PDB:1XTI) and H5N1 nucleoprotein (PDB: 2Q06)

**Figure 8:**
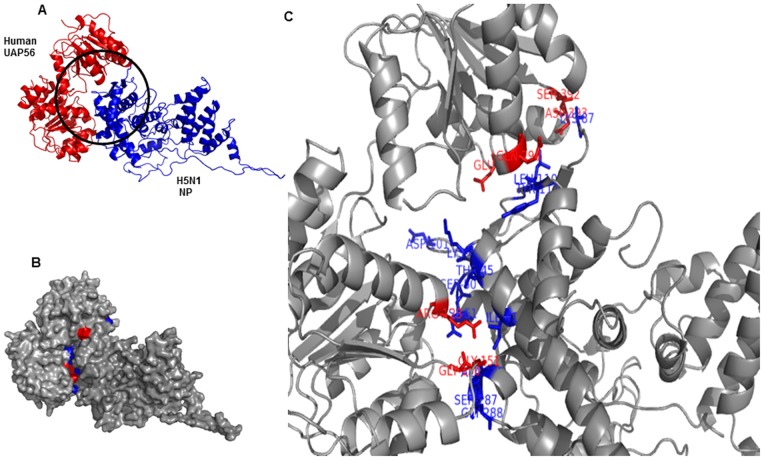
Molecular docking using crystal structures of human UAP56 (PDB: 1XTI) and H5N1 nucleoprotein (PDB: 2Q06). Figure 8A shows ribbon representation of docking between H5N1 NP and human UAP56. Both the molecules shows favorable and tight binding with binding energy of 35.42 kcal/mol, which interestingly seems stronger than the previous one (3ULH and 2Q06). There are several residues of 1XTI that interact with 2Q06; they are marked red: Arg123, Gly150, Gly151, Glu298, Glu366, Ser382, andAsp393. Residues of 2Q06 that interact with 1XTI are marked blue: Ile41, Thr45, Lys48, Ser50, Asp51, Lys87, Asp101, Leu108, Leu110, Tyr111, Ala286, Ser287, and Gly288. Magnified version of the interacting residues are shown in Figure 8C.

We conducted molecular docking studies using three-dimensional structure of human UAP56 (PDB: 1XTI) and H5N1 nucleoprotein (PDB: 2Q06). They interact favorably with a favorably binding energy of -35.42 kcal/mol, which interestingly seems stronger than the previous one (3ULH and 2Q06). There are several residues of 1XTI that interact with 2Q06; they are marked red: Arg123, Gly150, Gly151, Glu298, Glu366, Ser382, andAsp393. Residues of 2Q06 that interact with 1XTI are marked blue: Ile41, Thr45, Lys48, Ser50, Asp51, Lys87, Asp101, Leu108, Leu110, Tyr111, Ala286, Ser287, and Gly288. Figure 8 show how both protein interacts. In ribbon presentation, 1XTI is red and 2Q06 is blue (Figure 8A). In surface representation, 1XTI residues that interact with 2Q06 are red, and 2Q06 residues that interact with 1XTI are blue (Figure 8B). A magnified view showing interacting residues is shown in Figure 8C.

### H5N1 NP and UAP56 co-localize primarily in the nucleus of mammalian cells

**Figure 9:**
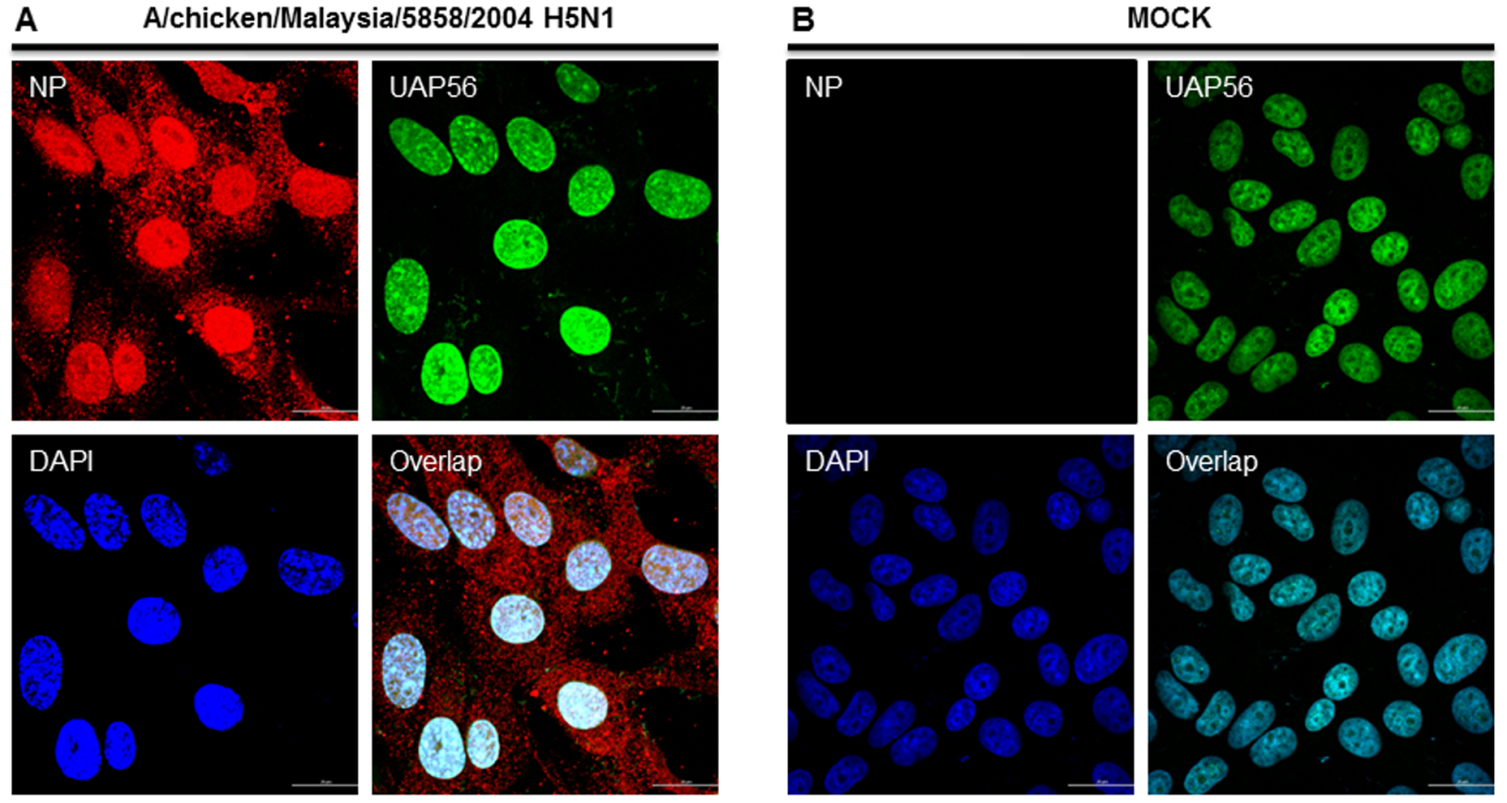
Co-localization of HPAI H5N1 NP from A/chicken/Malaysia/5858/2004 and UAP56 in nucleus of mammalian cells. ***A and B*** Vero cells were seeded onto cover slips in a 24 well plate at a density of 10^4^/well. The cells are then infected with influenza A/chicken/Malaysia/5858/2004 virus at a multiplicity of infection (MOI) of 5 (***For A only***). A low (4°C) temperature pre-incubation method was used to allow synchronized infection for 1 h in DMEM medium supplemented with 2% BSA (GIBCO). After 1 h incubation, the cells were washed with DMEM once and then grown with DMEM supplemented with 0.2% BSA and 1 µg/ml N-p-tosyl-1-phenyl alanine chloromethyl ketone (TPCK) (Sigma Aldrich). At 12 hour p.i., cells were fixed and processed for immunostaining. NP was stained using anti-NP monoclonal primary antibody and Alexa594 conjugated secondary antibody (Red). UAP56 was stained using mouse anti-UAP56 specific primary antibody (Santa Cruz) and Alexa488 conjugated secondary antibody (Green). Nuclei were stained with DAPI (Blue). ***A*** shows H5N1 infected cells whereas ***B*** shows control mock infected cells. Panels are labeled for their respective staining. Lower right panel in ***A*** shows primarily nuclear co-localization of NP and UAP56.

The distribution of UAP56 in influenza virus infected cells was not studied before. Thus, we investigated the cellular localization pattern of H5N1 NP in context to UAP56 in mammalian cells. First, we infected Vero cells with H5N1 (A/chicken/Malaysia/5858/2004) for 12 h, and an immunofluorescence staining was performed with specific antibodies. Results show that NP and cellular UAP56 co-localize primarily in the nucleus (Figure 9).

### siRNA mediated inhibition of UAP56 in H5N1 infected cells

**Figure 10:**
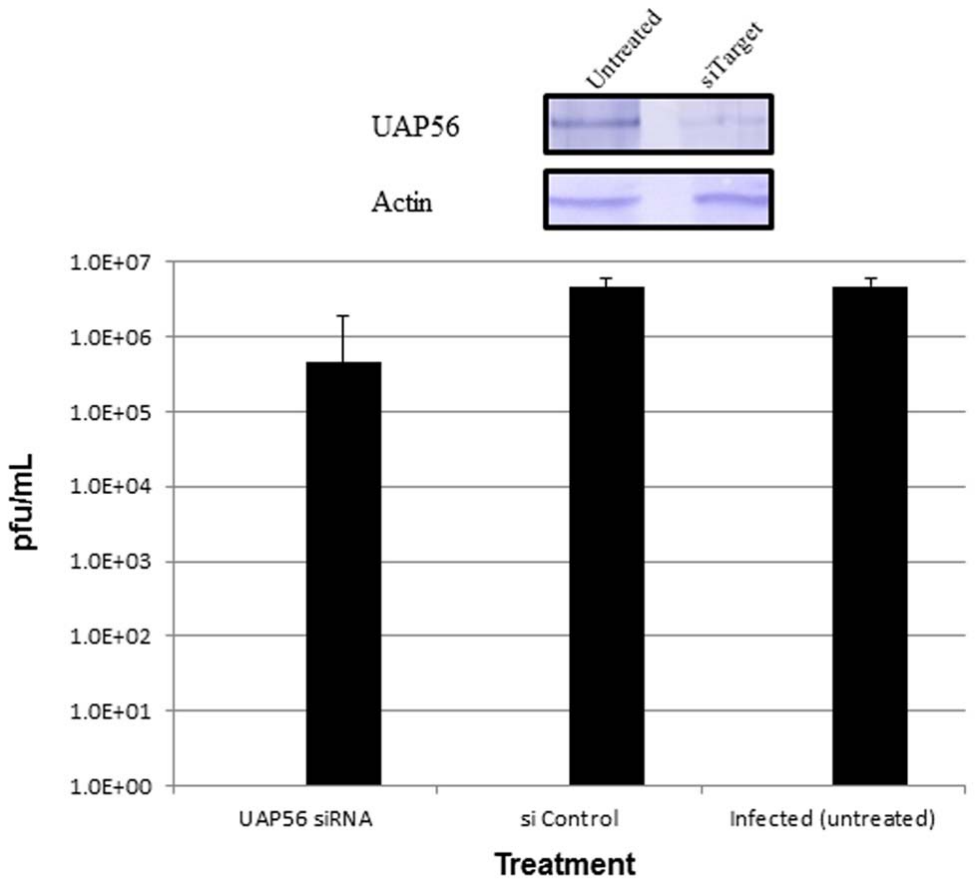
Knockdown of UAP56 impairs A/chicken/Malaysia/5858/2004 H5N1 virus growth. A549 cells were transfected with siRNA targeting UAP56 or with a non-targeting siRNA control as indicated. Fourty-eight hours later, depleted cells were infected with A/chicken/Malaysia/5858/2004 H5N1 at MOI of 5. Cells were harvested 48 hours after infection and viral titers were determined by plaque assays on Vero cells. Overall, knockdown of UAP56 decreased viral replication by nearly 10 fold when compared to non-treated. The mean ± SD of three independent experiments is shown.

Gene-specific small interfering RNAs (siRNAs) for UAP56 (DDX39B; 003805) provided as an On-Target Plus pools, which is a pool of four siRNAs targeting various sites in a single gene. The non-targeting siRNA 3 (siControl; D-001210) were provided as individual siRNA duplexes. All siRNAs were purchased from Dharmacon (Dharmacon Research Inc., Lafayette, CO) and transfected using Lipofectamine RNAiMAX (Invitrogen). Low-passage A549 cells were transfected with siRNA at a concentration of 100 nM for 48 hour according to the manufacturer's instructions. To study the effect of UAP56 knockdown on the replication of influenza virus, A/chicken/Malaysia/5858/2004 H5N1 strain was used to infect the siRNA treated A549 cells at an MOI of 5. Supernatants were collected 48 hour p.i. and the virus titers were determined by plaque assay on Vero cells. Overall, the plaque assay result showed significant reduction (nearly 10 fold) in virus yields in siRNA treated compared non-treated infected cells (Figure 10).

## Discussion

Export of mRNA from the nucleus to the cytoplasm is a carefully orchestrated multistep process. It requires RNA binding by export adaptor proteins that direct the transcript to the major mRNA export receptor TAP/NXF1, which then guides the mRNP through the nuclear pore complex (NPC) to the cytoplasm [9], [10], [11]. The cellular export adaptor protein is vital in bridging the transport as the TAP/NXF1 does not interact directly with RNA due to its low affinity for binding mRNAs. Aly/REF is one of the best characterized adaptors of TAP/NXF1. It binds to the 5′end of mRNA through an interaction with the cap binding protein CBP80 to form part of the TREX complex [12]. UAP56 is another TREX complex protein that recruits Aly/REF to the 5′ end of the RNA. The TREX complex has been shown to be involved in the export of both spliced and intronless RNA [13], [14]. UAP56 and Aly/REF are part of the multi protein TREX complex. While UAP56 was shown to be essential for mRNA export in both Drosophila and C. elegans, Aly/REF seems to be dispensable, suggesting the existence of additional mRNA export adaptors [15], [16].

In our report, we have shown the construction a novel chicken lung cDNA library which is fused inside the yeast vector pGADT7. As chickens are the primary host and chicken lungs the primary site of infection of the HPAI H5N1 virus; it was used to create the library. This will also mimic the same environment during infection; crucial to unravel novel host-pathogen interactions. We have provided evidence that H5N1 nucleoprotein, an essential influenza regulatory protein interacts with *Gallus gallus* Aly/REF; an important cellular adaptor protein in nucleocytoplasmic transport. NP-Aly/REF interaction was identified through a yeast two-hybrid screen and confirmed in transfected cells using mammalian two hybrid assay. Molecular docking studies suggest that this interaction also happens in human Aly/REF with NP from different subtype. We further proved this interaction using mammalian two hybrid again, but this time, using NP from A/New Jersey/1976/H1N1 and pandemic A/Malaysia/854/2009(H1N1) to interact with human Aly/REF. Both the NP from these subtypes showed strong interaction with human Aly/REF. It also observed that, these interactions were found to be localized primarily in the nucleus of infected cells (Vero and A549). Next, we asked a question whether, the knockdown of Aly/REF could impair the transport of viral mRNAs thus leading to reduction in overall viral titer in infected cells. In case of viral infection, in addition to cellular mRNAs, amounts of viral mRNAs have to be efficiently transported to the cytoplasm for translation. However, the silencing of Aly/REF in influenza infected cells did not significantly reduce the overall viral titer.

In a recent study by Sandri-Goldin, similar work has been done; whereby siRNA mediated knockdown of Aly/REF was done. Reduction in Aly/REF levels of greater than 80% had no discernible effect on the export of poly(A)^+^RNA in mock- or HSV-1 KOS-infected cells [14]. This finding coincides with ours and also by others such as Gatfield and Longman who has reported that Aly/REF is not essential for cellular mRNA export [15], [16]. In conjunction with this, if we look further at some of the studies done focusing on this phenomena, there are many other homologues of the NP such as the ICP27 of HSV-1, SM protein in Epstein-Barr virus and ORF57 in KSHV also interact with Aly/REF [14], [17], [18]. Aly/REF has also been reported to interact with UAP56, another component of the TREX complex, and UAP56 has been shown to recruit Aly/REF to mRNA [19], [20], [21]. Furthermore, it has been shown that the cytomegalovirus homologue of ICP27, termed UL69, interacts withUAP56 [22], and both Aly/REF and UAP56 were recruited to a KSHV mRNA, as well as other components of the TREX complex [23]. Sandri-Goldin has also reported the interaction between ICP27 and UAP56, whereby Aly/REF appears to stabilize this interaction. This intriguing finding may give rise to a possibility that, although the TREX complex may be essential to guide mRNA through the NPC in a 5′to 3′fashion [24], it is possible that the TREX complex can form with one of the components missing. This hypothesis would clearly explain on why the interaction between influenza A NP and host Aly/REF takes place. This phenomenon also coincides with several lines of evidence which suggests that in addition to roles in export, TREX proteins may play a role in preventing the rapid nuclear degradation that is observed with cDNA-derived transcripts and artificial noncoding RNAs [25, 26, 27, 28, 29, 30. 31]. Intronless cellular mRNAs contain sequences within their coding regions that increase nuclear stability and bind TREX complex proteins independent of splicing [32].

Knockdown of UAP56 has been reported to impair cellular mRNA export [19], [21], thus we investigated the role of UAP56 which is another component of the TREX complex, as UAP56 has been shown to recruit Aly/REF to mRNA. First, molecular docking studies using crystal structures from PDB shows that human UAP56 (1XTI) and H5N1 Nucleoprotein (2Q06) interacts favorably with a favorably binding energy of -35.42 kcal/mol, which seems stronger than the previous one Aly/REF (2ULH) and H5N1 Nucleoprotein (2Q06). Co-localization studies in Vero cells also shows that both these proteins also co-localizes in the nucleus at early infection similar to Aly/REF. Another intriguing finding is that, knockdown of UAP56 in A549 infected cells shows significant reduction in viral titer (about 10 fold).

## Materials and Methods

### Chicken lung (*Gallus gallus*) library construction

All animal studies were performed according to protocols approved by Animal Ethics committee of the Institute of Bioscience, University Putra Malaysia. Lungs were harvested from 4 weeks old specific pathogen free (SPF) white leghorn chicken. The lung tissues were kept at −80°C until used for total RNA extraction. Total RNA was extracted using Trizol® Reagent (Invitrogen, Carlsbad, USA). Purified RNA served as the template for first-and second-strand cDNA synthesis, using Make Your Own “Mate & Plate™” Library System (Clontech). This system utilized SMART™ cDNA synthesis technology, which enabled homologous recombination of cDNA library pool into yeast activation domain vector pGADT7-rec. Double-stranded cDNA less than 400 bp, was discarded using Chroma Spin™ TE-400 columns. For yeast transformations, competent yeast strain Y187 was prepared through high-efficiency polyethylene glycol (PEG)/LiAc-based method [33]. Purified double-stranded cDNA, in conjunction with 3 μg of linearized pGADT7-rec vector, and 200 μg of denatured Yeastmaker Carrier DNA (Clontech) were mixed and concurrently transformed into competent yeast Y187. Transformed yeast cells were treated with Yeast-Peptone-Dextrose (YPD) Plus medium(Clontech) to increase the transformation efficiency. Then, pelleted cells were resuspended in 15 ml of 0.9% (w/v) NaCl solution, prior to spreading on selection agar plates depleting Leucine (SD/-Leu). Survived colonies were pooled and kept in 1-ml aliquots for −80°C storage until use. The library titer was checked by spreading 100 μl of 10^−2^, 10^−3^, 10^−4^ diluted yeast cultures on SD/-Leu agar plates (diluted in 0.9% (w/v) NaCl solution), incubated for 4 days at 30°C. This library can potentially produce 6×10^7^yeast colony forming unit/ml. To check library complexity, an aliquot of the cDNA library was diluted to 10^−4^in 200 μl of 0.9% (w/v) NaCl solution. The sample was spread on a fresh SD/-Leu agar plate, incubated for 4 days at 30°C. Colony screening was conducted on 10 randomly picked colonies using 2× Terra™ PCR Direct Red Dye Premix (Clontech), in conjunction with 0.5 μM of forward and reverse Insert Check primers, in 20-μl PCR reactions. The PCR cycling parameters were as follows: 98°C for 2 min; 40 cycles of 98°C for 10 sec, 60°C for 15 sec, 68°C for 2.5 min; 68°C for 5 min; final hold at 10°C.

### Yeast Two-Hybrid Screening

Briefly, complete coding sequence of NP gene was cloned in-frame at the carboxy-terminus of GAL4 binding domain in plasmid pGBKT7 (Clontech), named pGBKT7-NP. Competent yeast strain Y2HGold was transformed with pGBKT7-NP, plated on synthetic dropout (SD) agar depleting tryptophan (SD/-Trp), and surviving yeast colony was inoculated in 5 ml YPDA broth at 30°C overnight. Then, a 1-ml library aliquot of a chicken cDNA library cloned in pGADT7 (Clontech) vector was used as prey mixed with the overnight culture, allowed for mating for a further 24 hours at 30°C at 45 rpm in a shaking incubator. The mixture was centrifuged and the resuspended pellets were spread onto low-stringency agar SD/-Leu/-Trp (double drop out) in the presence of Aureobasidin A (Aba) and X-α-gal, followed by high-stringency agar SD/-Leu/-Trp/-Ade/-His (Quadruple drop out, QDO) supplemented with Aba and X-α-gal. A total of 24 surviving blue colonies on QDO were subjected to PCR and sequencing. Sequences were then filtered and compared to the latest release of the GenBank database by using BLASTn (http://blast.ncbi.nlm.nih.gov/Blast.cgi). In the screen, we identified that H5N1 NP interacts with *Gallus gallus* THO complex 4 (THOC4) or Aly/REF, Gen Bank accession no. 363740920.

### Mammalian Two-Hybrid Assay

The mammalian Matchmaker two-hybrid assay kit 2, obtained from Clontech, was used to generate desired plasmid constructs. NP genes from all 3 subtypes, A/chicken/Malaysia/5858/2004 (pM_NP2004), A*/*NewJersey*/*1976*/*H1N1 (pM_NP1976) and pandemic A/Malaysia/854/2009(H1N1) (pM_NP2009) were subcloned into pM (GAL4 DNA-BD) vector while the *Gallus gallus* (pVP16_Aly Chick) and human (pVP16_Aly human) Aly/REF were cloned into the pVP16 (AD) cloning vectors (Clontech). HEK293T cells were grown in Dulbecco's modified Eagle's medium (DMEM) and transfected as described above. For mammalian two-hybrid assay, all three plasmids, pM_NP2004 (0.3 µg), pVP16_Aly Chick (0.3µg), and pG5SEAP reporter vector (0.3 µg) were co-transfected into HEK293T cells. This was also performed for both the other plasmids pM_NP1976 and pM_NP2009 with pVP16_Aly human in separate experiments. The cells were allowed to grow at 37°C in 5% CO_2_. After 48 h of transfection, the secreted alkaline phosphatase (SEAP) activity was detected using the Great EscAPe SEAP fluorescence detection kit (Clontech). Each experiment was done in triplicate, and three independent transfections were performed. Basal control (pM and pVP16), and controls for both NP and Aly/REF were included as described in Table 2. The data for basal controls were used for the conversion of SEAP activity to fold activation. The level of SEAP activity was detected in cell culture medium after 48 h of transfection using the manufacturer's instructions. Briefly, the cell culture medium was collected and centrifuged to remove any detached cells present in the cell medium. The fluorescence compound, 4-methylumbelliferylphosphate, was used as a substrate, and its fluorescence was determined by excitation at 360 nm and emission at 449 nm. A standard linear curve was obtained using the positive placental alkaline phosphatase. Paired student's t-test was used for statistical analysis.

### Immunofluorescence confocal microscopy and virus infection assay

All work involving influenza viruses were done at BSL-3 facility of the Institute of Bioscience, UPM, Malaysia. A549 (adenocarcinomic human alveolar basal epithelial) and Vero (African green monkey kidney) cells were seeded onto cover slips in a 24 well plate at a density of 10^4^/well. The cells are then infected with influenza A/chicken/Malaysia/5858/2004, A/New Jersey/1976/H1N1 or pandemic A/Malaysia/854/2009(H1N1) H5N1 virus at a multiplicity of infection (MOI) of 5. A low (4°C) temperature pre-incubation method was used to allow synchronized infection for 1 h in DMEM medium supplemented with 2% BSA (GIBCO). After 1 h incubation, the cells were washed with DMEM once and then grown with DMEM supplemented with 0.2% BSA and 1 µg/ml N-p-tosyl-1-phenyl alanine chloromethyl ketone (TPCK) (Sigma Aldrich). At 12 hour p.i., cells were fixed with 4% paraformaldehyde (PFA) for 30 min at room temperature. They were permeabilized with 0.5% Triton X-100 for 5 min at room temperature and blocked with PBS containing 2% bovine albumin. Immunostaining was performed using rabbit anti-NP (Abcam) and mouse anti-THOC 4 (Santa Cruz) and mouse anti-DDX39B (UAP56) (Santa Cruz) antibodies. Unbound antibodies were washed away with PBS and cells were incubated with Alexa 488 tagged goat anti-mouse antibodies and Alexa 594 tagged goat anti-rabbit. Nuclei were stained with DAPI. Photomicrographs were captured at 60X magnification using a laser scanning confocal microscope (C1si, Nikon, Tokyo, Japan). Images were processed using NIS Elements AR 4.0 software (Nikon, Tokyo, Japan).

### Molecular Docking

The three-dimensional structure of human Aly/REF (PDB: 3ULH), human UAP56 (PDB: 1XTI) and H5N1 nucleoprotein (PDB: 2Q06) is obtained from the Protein Data Bank (PDB). PatchDock algorithm was used to suggest a rigid docking model between Aly/REF (PDB: 3ULH) and influenza virus H5N1 nucleoprotein (H5N1 NP, PDB: 2Q06). This procedure was also used for UAP56 (PDB: 1XTI) and influenza virus H5N1 nucleoprotein (H5N1 NP, PDB: 2Q06). PatchDock maximizes surface shape complementarity of two interacting proteins while minimizing the steric clashes. Upon docking, PatchDock calculation resulted three-dimensional transformations of the protein's Cartesian coordinate, which were further refined and scored based on protein-protein interaction energy using FireDock algorithm. Complex structure with the lowest interaction energy was taken as the final model for further analysis.

### siRNA and viral plaque assay

Gene-specific small interfering RNAs (siRNAs) for Aly/REF (Thoc4; 012078) and UAP56 (DDX39B; 003805) provided as an On-Target Plus pools, which is a pool of four siRNAs targeting various sites in a single gene. The non-targeting siRNA 3 (siControl; D-001210) were provided as individual siRNA duplexes. All siRNAs were purchased from Dharmacon (Dharmacon Research Inc., Lafayette, CO). A549 cells at a density of 10^4^/well of a 24-well plate were transfected with 100 nM of the indicated siRNA for 48 h prior to infection with A/chicken/Malaysia/5858/2004 H5N1 strain at a MOI of 5. Supernatants were collected 48 hour p.i. and the virus titers were determined by plaque assay on Vero cells.

### Immunoblot assay analysis and antibodies

A549 cells were transfected with siRNA at a concentration of 100 nM for 24 h, 48 h and 96 h according to the manufacturer's instructions as described above, and the cell lysates were harvested and subjected to immunoblotting using anti-Aly/REF antibody Santa Cruz) to check for knockdown efficiency. Briefly, cells were lysed using a buffer (20 mM HEPES, pH 7.5, 150 mM NaCl, 1 mM EDTA, 10% glycerol, 1% Triton X-100) supplemented with protease-inhibitors (Sigma Aldrich) and the lysates were subject to SDS PAGE. Beta actin (GenTex) was used as loading control.

## Supporting Information

Figure S1 **Confirmation of interaction between H5N1 NP and Aly/REF in mammalian cells transfected with NP expressing plasmid.** A549 cells were transfected with pcDNA3.1-NP and pcDNA3.1-Aly/REF plasmids alone or in combination, 48 hours post-transfection cells were harvested and IP was setup using anti-NP-specific antibody and anti-Aly/REF specific antibody. Lanes 2 and 4 show co-IP of Aly/REF with NP and vice-versa.
(TIF)

Figure S2 **Yeast two hybrid screening and identification of** ***Gallus gallus*** **Aly/REF as interacting partner of H5N1 Nucleoprotein.** Coding sequence of NP gene was cloned in-frame at the carboxy-terminus of GAL4 binding domain in plasmid pGBKT7 (Clontech), named pGBKT7-NP. Competent yeast strain Y2HGold was transformed with pGBKT7-NP, plated on synthetic dropout (SD) agar depleting tryptophan (SD/-Trp), and surviving yeast colony was inoculated in 5 ml YPDA broth at 30°C overnight. Then, a 1-ml library aliquot of a chicken cDNA library cloned in pGADT7 (Clontech) vector was used as prey mixed with the overnight culture, allowed for mating for a further 24 hours at 30°C at 45 rpm in a shaking incubator. The mixture was centrifuged and the resuspended pellets were spread onto low-stringency agar SD/-Leu/-Trp (double drop out) in the presence of Aureobasidin A (Aba) and X-α-gal, followed by high-stringency agar SD/-Leu/-Trp/-Ade/-His (Quadruple drop out, QDO) supplemented with Aba and X-α-gal. A total of 24 surviving blue colonies on QDO were subjected to PCR and sequencing. ***Panel A*** shows the overall result of the screening. ***B*** shows an example of a SD/-Leu/-Trp (double drop out) plate in the presence of Aureobasidin A (Aba) and X-α-gal. Note the white arrows in ***B*** indicating weak interaction. ***Panel C and D*** shows an example of high-stringency agar plate SD/-Leu/-Trp/-Ade/-His (Quadruple drop out, QDO) supplemented with Aba and X-α-gal. White arrows in ***Panel D*** indicate the colonies previously from DDO did not survive. Surviving colonies in QDO plates are sequenced and identified as Aly/REF.
(DOCX)
