# Supplementary figures and images for: Highly Pathogenic Avian Influenza Virus Nucleoprotein Interacts with TREX Complex Adaptor Protein Aly/REF
Source: PLoS One. 2013 Sep 20;8(9):e72429. doi: 10.1371/journal.pone.0072429 (PMC3779218; doi:10.1371/journal.pone.0072429)

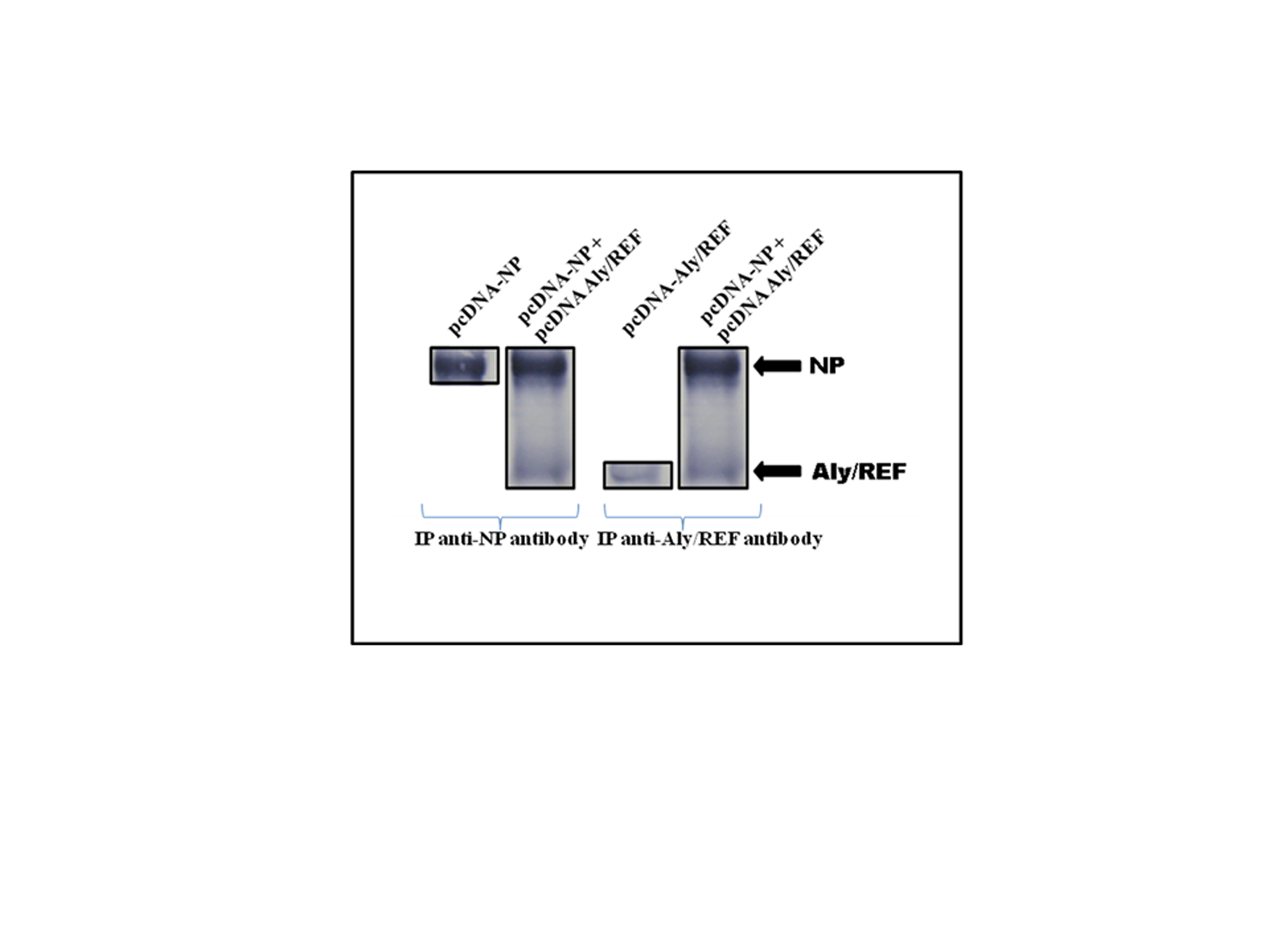

Supplement: Figure S1 — Confirmation of interaction between H5N1 NP and Aly/REF in mammalian cells transfected with NP expressing plasmid. A549 cells were transfected with pcDNA3.1-NP and pcDNA3.1-Aly/REF plasmids alone or in combination, 48 hours post-transfection cells were harvested and IP was setup using anti-NP-specific antibody and anti-Aly/REF specific antibody. Lanes 2 and 4 show co-IP of Aly/REF with NP and vice-versa. (TIF) [file pone.0072429.s001.tif]
