# Supplementary material for: Highly Pathogenic Avian Influenza Virus Nucleoprotein Interacts with TREX Complex Adaptor Protein Aly/REF
Source: PLoS One. 2013 Sep 20;8(9):e72429. doi: 10.1371/journal.pone.0072429 (PMC3779218; doi:10.1371/journal.pone.0072429)
Supplement: Figure S2 — Yeast two hybrid screening and identification of Gallus gallus Aly/REF as interacting partner of H5N1 Nucleoprotein. Coding sequence of NP gene was cloned in-frame at the carboxy-terminus of GAL4 binding domain in plasmid pGBKT7 (Clontech), named pGBKT7-NP. Competent yeast strain Y2HGold was transformed with pGBKT7-NP, plated on synthetic dropout (SD) agar depleting tryptophan (SD/-Trp), and surviving yeast colony was inoculated in 5 ml YPDA broth at 30°C overnight. Then, a 1-ml library aliquot of a chicken cDNA library cloned in pGADT7 (Clontech) vector was used as prey mixed with the overnight culture, allowed for mating for a further 24 hours at 30°C at 45 rpm in a shaking incubator. The mixture was centrifuged and the resuspended pellets were spread onto low-stringency agar SD/-Leu/-Trp (double drop out) in the presence of Aureobasidin A (Aba) and X-α-gal, followed by high-stringency agar SD/-Leu/-Trp/-Ade/-His (Quadruple drop out, QDO) supplemented with Aba and X-α-gal. A total of 24 surviving blue colonies on QDO were subjected to PCR and sequencing. Panel A shows the overall result of the screening. B shows an example of a SD/-Leu/-Trp (double drop out) plate in the presence of Aureobasidin A (Aba) and X-α-gal. Note the white arrows in B indicating weak interaction. Panel C and D shows an example of high-stringency agar plate SD/-Leu/-Trp/-Ade/-His (Quadruple drop out, QDO) supplemented with Aba and X-α-gal. White arrows in Panel D indicate the colonies previously from DDO did not survive. Surviving colonies in QDO plates are sequenced and identified as Aly/REF. (DOCX) [file pone.0072429.s002.docx]

| **Sample** | **Selective Agar Plate** | **Distinct 2mm colonies** | **Color** |
| --- | --- | --- | --- |
| Bait + candidate prey | DDO/X | Yes | Blue |
|  | QDO/X/A | Yes | Blue |
| Empty pGBKT7 + candidate prey | DDO/X | Yes | White |
|  | QDO/X/A | No | N/A |

A

**With Bait (H5N1 NP) and candidate prey (chicken lung cDNA library)**

B


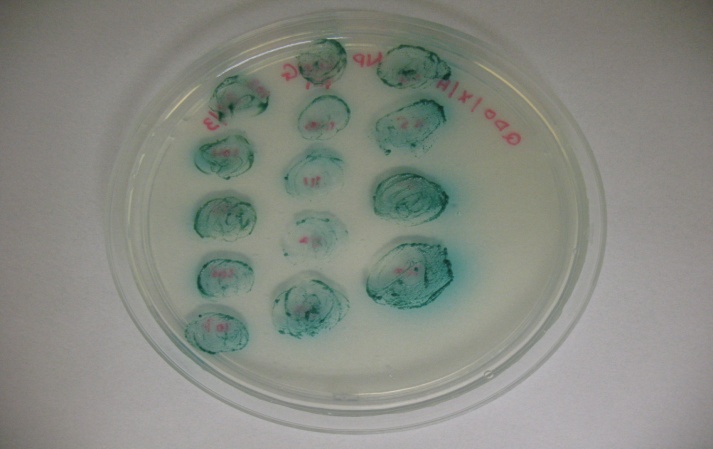


D

C


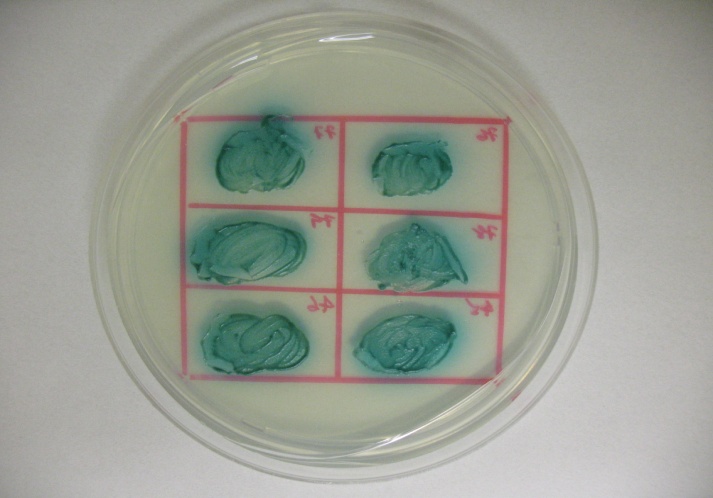

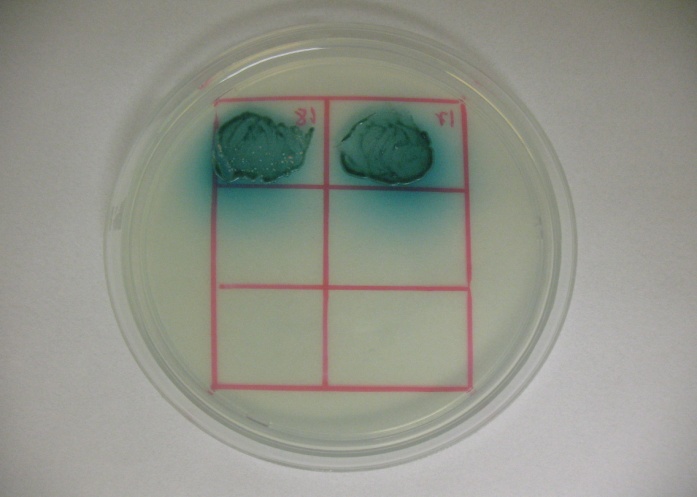


C
